# eldBETA: A Large Eldercare-oriented Benchmark Database of SSVEP-BCI for the Aging Population

**DOI:** 10.1038/s41597-022-01372-9

**Published:** 2022-05-31

**Authors:** Bingchuan Liu, Yijun Wang, Xiaorong Gao, Xiaogang Chen

**Affiliations:** 1 Department of Biomedical Engineering, School of Medicine, Tsinghua University, Beijing, 100084 China; 2 State Key Laboratory on Integrated Optoelectronics, Institute of Semiconductors, Chinese Academy of Sciences, Beijing, 100083 China; 3 Institute of Biomedical Engineering, Chinese Academy of Medical Sciences and Peking Union Medical College, Tianjin, 300192 China

**Keywords:** Biomedical engineering, Health care

## Abstract

Global population aging poses an unprecedented challenge and calls for a rising effort in eldercare and healthcare. Steady-state visual evoked potential based brain-computer interface (SSVEP-BCI) boasts its high transfer rate and shows great promise in real-world applications to support aging. Public database is critically important for designing the SSVEP-BCI systems. However, the SSVEP-BCI database tailored for the elder is scarce in existing studies. Therefore, in this study, we present a large **eld**ercare-oriented **BE**nchmark database of SSVEP-BCI for **T**he **A**ging population (eldBETA). The eldBETA database consisted of the 64-channel electroencephalogram (EEG) from 100 elder participants, each of whom performed seven blocks of 9-target SSVEP-BCI task. The quality and characteristics of the eldBETA database were validated by a series of analyses followed by a classification analysis of thirteen frequency recognition methods. We expect that the eldBETA database would provide a substrate for the design and optimization of the BCI systems intended for the elders. The eldBETA database is open-access for research and can be downloaded from the website 10.6084/m9.figshare.18032669.

## Abstract (summary)

**Table:** 

Measurement(s)	Steady-state visual evoked potential (SSVEP)
Technology Type(s)	Electroencephalography (EEG)
Factor Type(s)	Elder population
Sample Characteristic - Organism	Homo sapiens
Sample Characteristic - Environment	Electromagnetic shielding room

## Background & Summary

Aging population grows at an accelerating pace worldwide^1–5^. Over the past 180 years, the record life expectancy for humans has steadily increased by 2.5 years per decade and people have longer lives than ever before^4,5^. The longer lives are caused by the postponement of mortality, and most residents born in this century and in countries with high life expectancies will celebrate their 100th birthdays^2,3^. As a result of the longer livers and low fertility, the world is confronting an aging population, and one in five of the people in the world is projected to be elder citizens (60 years or above) by 2050^1,6^. This situation is more grave in parts of the world, i.e., Europe and China, where one in four people is projected to be the elder by 2050^1,6,7^. The global challenge of aging motivates the rising need of eldercare and technological support for the elder^7–9^.

Brain-computer interface (BCI) provides a direct path between the brain and external device for alternative and augmentative communication^10,11^, which suits the need for the eldercare. Among the BCI paradigms, steady-state visual evoked potential based BCI (SSVEP-BCI)^12,13^ has received increasing attention due to its noninvasiveness, high information transfer rate (ITR)^13,14^, zero calibration^15,16^, and low BCI-illiterate rate^17^. The high transfer rate of SSVEP-BCI is attributable to the high signal-to-noise ratio (SNR) of a typical brain response, i.e., SSVEP^18^, which is a frequency-tagged brain response elicited by periodic visual stimuli. The merits of SSVEP-BCI make it a prime candidate for real-world applications in eldercare and healthcare, e.g., brain-controlled wheelchair^19,20^, exoskeleton^21,22^, assistive robots^23,24^, and emergency call^25^.

Public databases play a vital role in designing and optimizing pattern recognition systems in real-world applications. For instance, in the field of computer vision, the open database of ImageNet^26^ has a far-reaching impact on the renewed flourish of artificial intelligence (AI). For the field of SSVEP-BCI, a number of studies in the literature contribute to the efforts in curating public databases^17,27–30^ and sharing relevant data^31–34^. Specifically for the public SSVEP-BCI database, however, the vast majority of participants are young adults, and little attention so far has been paid to the elder. For the elderly population, a database targeted at this community could provide an opportunity to design a BCI system better suited for the eldercare applications, considering age-related differences existent in the BCI performance^35–42^. In particular, for SSVEP-BCI, previous studies reported that the elderly people achieved significantly lower ITR and accuracy than the younger participants^35,40,41^. The deteriorated BCI performance is attributable to the signal profile of decreased SSVEP amplitude associated with the age-related changes^38,42–44^, and physiologically further related to degradation in crystalline lens^45^, influence in retinal and central visual pathways^43^, and cell loss in visual cortex due to senescence^46^. Since there is a substantial distinction in SSVEP between the younger and older population, it is worthwhile to leverage the signal profile to develop high-speed SSVEP-BCI systems for eldercare applications.

To meet the demand for the open database designated for the elder, here we present a large **eld**ercare-oriented **BE**nchmark database of SSVEP-BCI for **T**he **A**ging population (eldBETA) in this paper. The eldBETA database features a large number of participants, i.e., 100 in the study, with old age of average 63 years, and up to 81 years. Also, the database of electroencephalogram (EEG) was collected under a laboratory experimental protocol, which provided the database with a signal quality of gold standard in designing the practical applications tailored for the elderly community. In the experimental protocol, seven blocks of 9-target SSVEP-BCI tasks were performed for each participant, while 64 channels of continuous EEG including 5-s SSVEP for each trial were recorded. The data records were validated by signal profiles followed by SNR analysis and BCI quotient to demonstrate the quality and distribution of the database. The utility of the database was validated by the classification analysis, showing the elder could achieve an average ITR up to approximately 150 bpm by supervised methods and an average ITR up to approximately 60 bpm by training-free methods. Additionally, the fractal characteristics of the elderly population were validated by power-law analysis. In sum, the eldBETA database offers an opportunity to facilitate the development of methods and systems on BCI healthcare targeted at the elder, and contributes to extending our understanding of BCI technology in the era of the aging population.

## Methods

### Participants

This study recruited elderly volunteers with ages greater than 50 years old. One hundred participants (33 males and 67 females) took part in this study. The age of the participants ranged from 52 to 81 with an average of $63.17\pm 6.05$ (mean ± standard deviation). All the participants had a normal or corrected-to-normal vision. The participants were instructed to be familiarized with experimental protocol and gave full written consent before the experiment. The experimental protocol was under the declaration of Helsinki and approved by the institutional review board of Tsinghua University (NO. 20210032).

### Brain speller

**Fig. 1:**
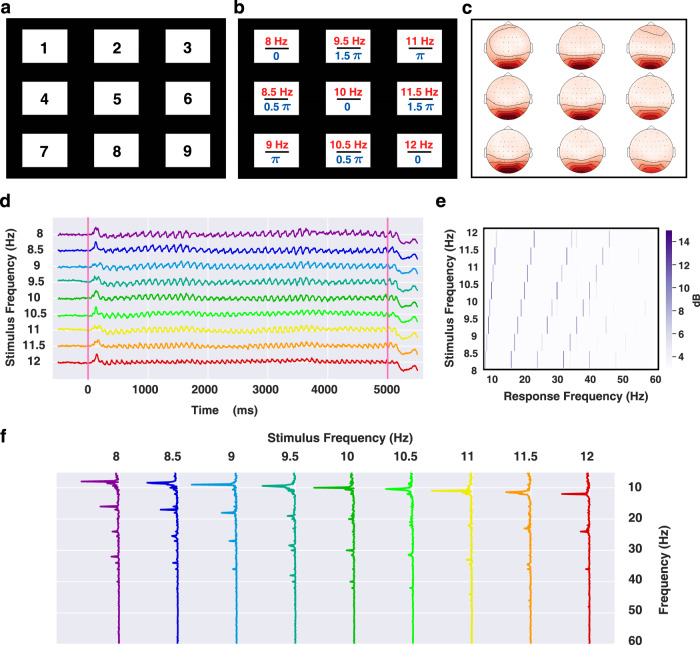
The virtual keyboard of a brain speller and the signal profile of SSVEP responses. (**a**) The layout of the virtual keyboard for dialing with 9 digits (1∼9). (**b**) The stimulus frequency (red) and initial phase (blue) encoded for each target by the JFPM method. (**c**) The topographic maps of the spectral amplitude at the fundamental frequency corresponding to each target on the speller. (**d**) The temporal profile of the grand-average SSVEPs. The oscillations for each stimulus frequency were visible during the visual stimulation marked by the pink lines. (**e**) The relationship between stimulus frequency and response frequency in the spectrum for the narrow-band SNR. The response frequency of SSVEP increases linearly with stimulus frequency with respect to fundamentals and harmonics. (**f**) The spectral profile of the grand-average SSVEPs. The spectral peaks were prominent at each stimulus frequency and were observable up to the 4th harmonics. (Best view in color).

In this study, a 9-target brain speller of SSVEP-BCI was developed for the elder participants. As illustrated in Fig. 1(a), the 9 targets were aligned in a 3 × 3 matrix, which was visually presented on the screen of an LCD monitor (refresh rate: 60 Hz; resolution: $1920\times 1080$ pixels). Each target had a dimension of 168 × 125 pixels and a digit character (a number from 1 to 9) lay at its center. The horizontal interval between adjacent targets was 100 pixels, and the vertical interval was 70 pixels. For the brain speller, the targets were encoded by joint frequency and phase modulation (JFPM)^13^, and the frequency and initial phase corresponding to the *i*-th row and the *j*-th column were obtained by

1 $$\begin{array}{l}{f}_{i,j}={f}_{0}+[3(j-1)+i-1]\cdot \Delta f\\ {\Phi }_{i,j}{=\Phi }_{0}+[3(j-1)+i-1]\cdot \Delta \Phi \end{array}$$

where ${f}_{0}$ (${\Phi }_{0}$) denotes the lower limit of stimulus frequency (initial phase), and $\Delta f$ ($\Delta \Phi$) denotes the frequency interval (phase interval). In this study, the frequency information was set as ${f}_{0}=8$ Hz, $\Delta f=0.5$ Hz, and the initial phase information was set as ${\Phi }_{0}=0$, $\Delta \Phi =0.5\pi$. The details of the encoded information can be found in Fig. 1(b).

The encoded information was used for generating visual flickers to evoke SSVEPs. In the implementation, a sampled sinusoidal stimulation method^47^ was employed under the environment of Psychophysics Toolbox^48^ in MATLAB (MathWorks, Inc.). Specifically, the grayscale value of the stimulus sequence for each flicker has the form

2 $$s(f,\phi ,i)=\frac{1}{2}\{1+{\rm{s}}{\rm{i}}{\rm{n}}[2\pi f(i/{f}_{r})+\phi ]\}$$

where ${f}_{r}$ denotes the refresh rate of the screen, and *i* denotes the current frame index of the sequence. The grayscale values of 0 and 1 denote the lowest and highest luminance of the screen, respectively.

### Experimental procedure

In this study, each participant took part in seven blocks of online SSVEP-BCI task, in which multi-channel EEG were decoded in real time after a target was selected. Each block consisted of nine trials that corresponded to the nine targets on the speller. The timeline of each trial was as follows. At the beginning, one of the targets was cued and the border of the target was colored green for 4 s. The order of cues was randomized in the experiment and each target was cued once in a block. Followed by the cue, participants directed their attention to the target. Then all targets started to flicker simultaneously for 5 s and participants were instructed to gaze at the center of the target. After the flickering process, there was 1-s rest time for the online feedback, which was presented as a red rectangle covering the target. A training-free method of FBCCA^16^ was adopted in the online processing. At the end of each block, participants were encouraged to have a brief rest to avoid visual fatigue. The duration of break time was controlled by the participant, generally ranging from 1 min to 5 min with an average of 3 min.

### Data acquisition

This study recorded 64-channel EEG data during the SSVEP-BCI task. The data were acquired using SynAmps2 (Neuroscan Inc., Charlotte, USA) at a sampling rate of 1000 Hz. The recorded EEG were well synchronised to the triggers events of the SSVEP task by means of a parallel port, which is a gold standard in synchronisation in BCI systems. The montage was aligned according to the international 10-20 system and the vertex Cz was used as the reference of the montage. The impedances of all channels were kept below 20 k$\Omega$ before the experiment. Nine parietal and occipital channels, i.e., Pz, PO3/4, PO5/6, POz, Oz and O1/2, were used for the online processing. The data were collected in the electromagnetic shielding room. To suppress noise, a build-in notch filter was employed to remove the power-line interference.

### Data preprocessing

The recorded offline data were preprocessed for storage and technical validation. To preserve broadband spectral properties, no filtering procedure was applied in the preprocessing. Continuous EEG data were extracted into EEG epochs, which comprised 0.5-s recordings before visual stimulation, 5-s responses of stimulation (SSVEP) and 0.5-s recordings after the stimulation. Then a downsampling procedure from 1000 Hz to 250 Hz was applied to the epochs.

### Evaluation metrics

#### Signal-to-noise ratio (SNR)

The responses of SSVEP are well characterized in the spectral domain, in which signal-to-noise ratio (SNR) can be quantitatively measured. Here, a wide-band SNR was employed for it could better evaluate the level of harmonics, noise and the perspective BCI performance^29^. The SNR of SSVEP (in decibels, dB) is defined as follows^29^

3 $$SNR=10{{\rm{\log }}}_{10}\frac{{\sum }_{k=1}^{k={N}_{h}}P(k\cdot {f}_{n})}{{\sum }_{f=0}^{f={f}_{s}/2}P(f)-{\sum }_{k=1}^{k={N}_{h}}P(k\cdot {f}_{n})}$$

where ${f}_{n}$ denotes the stimulus frequency, $P(f)$ denotes the power spectral density for the frequency $f$, and ${N}_{h}$ denotes the number of harmonics. For ${f}_{n}$ in the low-frequency band, ${N}_{h}$ is usually set 5^29^.

#### Information transfer rate (ITR)

As a widely used metric in the BCI community, the ITR measures the performance of participants as well as classification algorithms by means of information theory. Taking into account the number of targets (*M*) and the average target selection time (*T* in seconds), the classification accuracy (*P*) can be converted to the ITR (in bits per min, bpm), which is defined as follows^10^

4 $$ITR=60\cdot \left({{\rm{\log }}}_{2}M+P{{\rm{\log }}}_{2}P+(1-P){{\rm{\log }}}_{2}\frac{1-P}{M-1}\right)/T$$

Note that *T* includes the gaze time (e.g., 5 s) and the overall gaze shift time. For offline analysis, a gaze shift time of 0.5 s^13^ is usually set for validation and model comparison.

#### BCI quotient (BCIQ)

The metric of BCI quotient (BCIQ) provides a means to characterize the individual difference in the participant’s potential of leveraging the SSVEP-BCI^29^. The BCIQ estimates the quantile of a participant in SNR according to the scaling procedure of intelligence quotient^49^. Thus this metric is defined at the level of population, different from the level of single trial for SNR and the level of block for ITR. Derived from the SNR, the BCIQ is defined as follows^29^

5 $$BCIQ=15\cdot \frac{SNR-\mu }{\sigma }+100$$

where *μ* and *σ* denote the mean and standard deviation of the SNR by participant, respectively. In this study, $\mu =-11.87$ and $\sigma =2.86$.

## Data Records

**Fig. 2:**
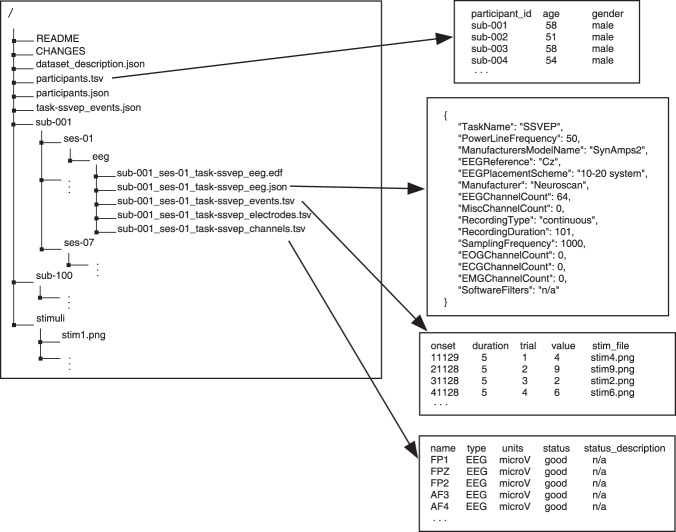
A preview of raw EEG data records of the eldBETA database. The data records were curated according to the EEG Brain Imaging Data Structure (EEG-BIDS)^50^. The raw EEG were stored in the European data format (“.edf”). The prefix “sub” denotes the participant and “ses” denotes the block (session).

This proposed database contained the data records of EEG from 100 participants, which were de-identified and indexed as S1∼S100. For each participant, continuous EEG data records in the form of EEG Brain Imaging Data Structure (EEG-BIDS)^50^ were provided. We also provided the associated records of epoch data that were stored in “.mat” structure array from MATLAB. The structure array was composed of the EEG data (“EEG”) and its associated supplementary information (“Suppl_info”) as its fields. The raw data can be found at Figshare and stored in “The eldBETA database” repository at the website^51^ 10.6084/m9.figshare.18032669. For convenient access to the data records, the database has an alternative source for storage at http://bci.med.tsinghua.edu.cn/download.html. Two types of EEG data, i.e., EEG epochs and raw EEG were provided for researchers to facilitate diverse research purposes. The EEG epochs were the EEG data with the data processing and stored as 4-dimensional matrices (channel × time point × condition × block). The names and locations of the channel dimension were given in the supplementary information. For the dimension of time point, the epochs had a length of 6 s, which included 0.5 s before the stimulus onset, 5 s during the stimulation (SSVEPs) and 0.5 s after the stimulus offset. For the dimension of condition, each index of the array corresponded to a stimulus frequency and the details were listed in Table 1. Different from the epoch data, the raw EEG provided continuous EEG that were converted by EEGLAB^52^. According to EEG-BIDS^50^, each block of raw EEG data was curated in a folder (e.g., “ses-01”), in which the EEG were stored in “.edf” files and the associated information can be found in “.tsv” and “.json” files. A preview of the raw data record is illustrated in Fig. 2.

**Table 1:** The stimulus frequency corresponding to the index of condition in the data records.

Index	1	2	3	4	5	6	7	8	9
Stimulus frequency	8 Hz	9.5 Hz	11 Hz	8.5 Hz	10 Hz	11.5 Hz	9 Hz	10.5 Hz	12 Hz

The “Suppl_info” field of the epoch record provided basic information about personal statistics and experimental protocol. The personal statistics included the age, gender, BCIQ and SNR with respect to each participant. The experimental protocol included channel location (“Channel”), stimulus frequency (“Frequency”), initial stimulus phase (“Phase”) and sampling rate (“Srate”). The channel location was represented by a 64 × 4 cell array. The first column and the fourth column denoted the channel index and channel name, respectively. The second column and the third column denoted the channel location in polar coordinates, i.e., degree and radius, respectively. The initial stimulus phase was given in radius. The sampling rate of the epoch data was denoted by “Srate”. A detailed data structure of the records was summarized in Table 2.

**Table 2:** The data structure and the content of epoch data.

Field	Sub-field	Data format
EEG	Epoch	4-dimensional matrix
Suppl_info	Participant_id	String
	Age	Integer
	Gender	String
	Channel	64 × 4 cell array
	Frequency	1 × 9 double
	Phase	1 × 9 double
	BCIQ	Double
	SNR	Double
	Srate	Double

## Technical Validation

### Signal profile and SNR analysis

We initially validated the data records by the visual inspections of temporal, spectral and spatial characteristics. A grand average was performed across blocks and participants to enhance the SNR of SSVEPs. Oz was chosen as the representative channel for the temporal and spectral visual inspections, which were shown in Fig. 1(d),(f). For each stimulus frequency, the oscillations were visible in the temporal progression of SSVEPs during visual stimulation. Rhythms in the temporal domain corresponded to the spectral domain, as illustrated in Fig. 1(f), where there was a prominent peak at each stimulus frequency. Apart from the fundamental frequency, the spectral peaks were observable up to the 4th harmonics. Figure 1(e) further illustrated the relationship between stimulus frequency and response frequency in the spectrum, where the narrow-band SNR was calculated for each spectral bin^13^. In line with the previous studies^13,27,29^, Fig. 1(e) showcased a predominant frequency-following response, where the response frequency of SSVEP increases linearly with the stimulus frequencies for the fundamentals and harmonics. For all channels at the scalp, the spectral amplitudes at each stimulus frequency were then topographically mapped, as depicted in Fig. 1(c). Each map corresponded to a target on the brain speller and the result showed that SSVEPs were dominantly distributed in the parietal and occipital regions across targets. Taken together, the visual inspections verified the hallmark features of SSVEPs.

**Fig. 3:**
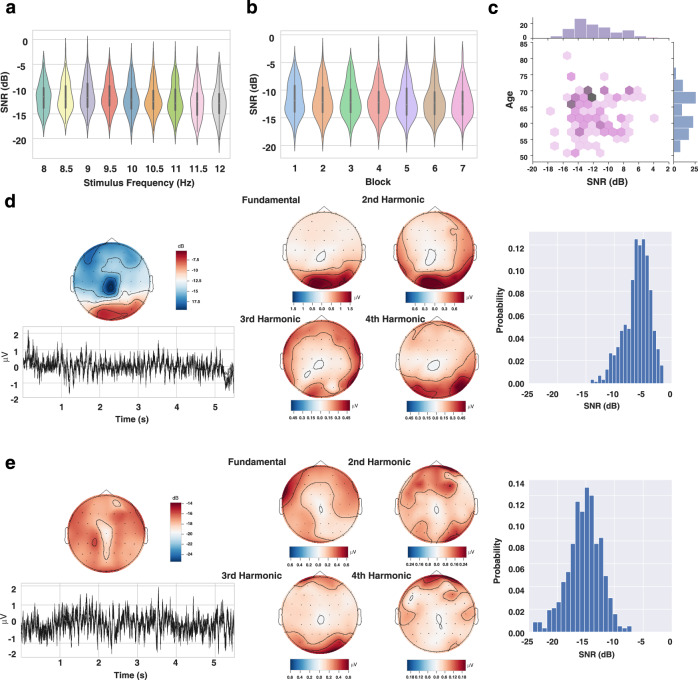
The SNR profile of the data records. (**a**) The change in SNRs with respect to the stimulus frequency. (**b**) The change in SNRs with respect to the block number in the experiment. (**c**) The joint and marginal distributions of SNRs and ages. (**d**) An overview of a representative participant with high SNR. In this case, the SNRs were distributed toward high values and there was a pattern of dense distribution in the occipital region. (**e**) An overview of a representative participant with low SNR. In this case, the histogram has a distribution of low SNRs and no specific pattern could be observed in the topographic maps. In (**d**) and (**e**), upper left panel: topographic maps of SNR; bottom left panel: temporal waves of average SSVEPs; middle panel: topographic maps of spectral amplitude at the fundamental frequency, 2nd, 3rd and 4th harmonics; right panel: histogram of SNRs. Note that (**a**), (**b**) and (**c**) are from all participants and all trials, while (**d**) and (**e**) are from all trials for a specific participant. (Best view in color).

To validate the SNR feature of the data records, the SNRs were calculated according to Eq. (3). Specifically, the nine channels (Pz, PO3/4, PO5/6, POz, Oz and O1/2) were assessed and the resultant values were then averaged by channels. Also, a data length of 4 s, including the 0.5s∼4.5s of the SSVEP, was used for analysis. The changes in SNRs with respect to the stimulus frequency and blocks were then visualized. As illustrated in Fig. 3(a), there was an overall tendency of decline in SNR as the stimulus frequency increased ($r=-0.1472$, $p < 0.001$), which was in line with the previous study^29^. Figure 3(b) delineated the change in SNR with blocks, showing that the SNRs of the data records were in a slightly declining tendency, though the decline was not statistically significant ($r=-0.0547$, $p=0.148$). At the individual level, the distribution of SNRs together with ages was presented in Fig. 3(c), from which a dense distribution around 70 years and −13 dB could be identified. Furthermore, two types of data records in terms of SNR were exemplified and the signal profile of SNR topographic maps, averaged temporal waves, spectral topographic maps (at fundamental and harmonic frequencies) and SNR histograms were shown. Figure 3(d,e) illustrate the signal profile of a representative participant with high SNR (S55) and low SNR (S79), respectively. For the participant with high SNR (Fig. 3(d)), the SNR maps (upper left panel) as well as spectral maps (middle panel) were characterized by a pattern of dense distribution in the occipital region, whereas there were no specific patterns for the participant with low SNR (Fig. 3(e)).

**Fig. 4:**
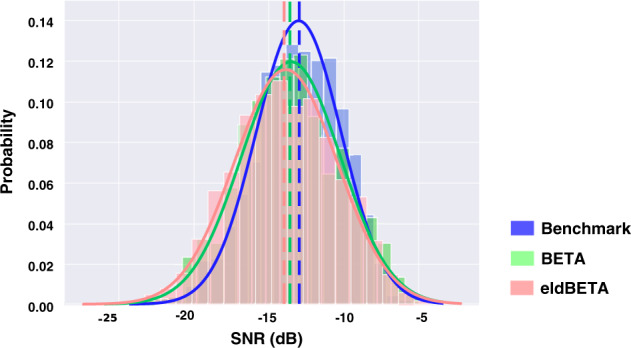
The histograms of SNRs from the eldBETA, the BETA and the Benchmark databases. The curve denotes the fitted normal probability density function (PDF) of the distribution. The dashed line denotes the mean of the distribution. It is noticeable that eldBETA database has a lower SNR than the BETA database and the Benchmark database. (Best view in color).

We then compared the SNR of the database with the other two public SSVEP-BCI databases, i.e., the Benchmark database^27^ and the BETA database^29^. For a fair comparison, SSVEPs from 0 to 3 s and a subset of stimulus frequencies (8 Hz, 9 Hz, 10 Hz, 11 Hz, and 12 Hz) were selected for analysis for the three databases. For the BETA database, participants from S16 to S70 were selected. Trials from the subset were band-pass filtered between 3 Hz and 100 Hz for the three databases, and were then padded with 2-s zeros^29^. This procedure was applied to the nine channels (Pz, PO3/4, PO5/6, POz, Oz and O1/2) and the histogram of the SNRs from the three databases were illustrated in Fig. 4. The result showed that the SNRs of the data records ($-13.902\pm 3.94$ dB) were significantly lower than that of the BETA database ($-13.56\pm 4.09$ dB, $p < 0.001$), and also than that of the Benchmark database ($-13.043\pm 3.84$ dB, $p < 0.001$). In Supplementary Figure 1, we further illustrated the topographic maps of SNRs and SSVEP amplitudes for aging and young participants, in which the aging participants were from the eldBETA database and the young participants were from the Benchmark database. The result showed that SSVEP signals for aging participants had substantially lower SNR and SSVEP amplitude than that for young participants. The deterioration of the SSVEP signal profile for the elder participants is in line with the previous studies^38,42–44^.

### Classification analysis

To validate the utility of the data records, this study conducted a classification analysis by benchmarking thirteen frequency recognition methods, including seven supervised methods and six training-free methods. Specifically, for each trial, a sliding window of data length (${N}_{p}$) from 0.1 s to 5 s with an interval of 0.1 s was used for analysis. The onset and boundary of the sliding window were set at $[{T}_{s}+d,{T}_{s}+d+{N}_{p}]$, where ${T}_{s}$ denotes the time point when visual stimulation starts. *d* denotes the latency of the visual system and conventionally *d* is set 140 ms^13^. Classification accuracy and ITR were used as evaluation metrics. For the metric of ITR, a gaze shift time of 0.5 s was used for analysis^16,27^.

**Fig. 5:**
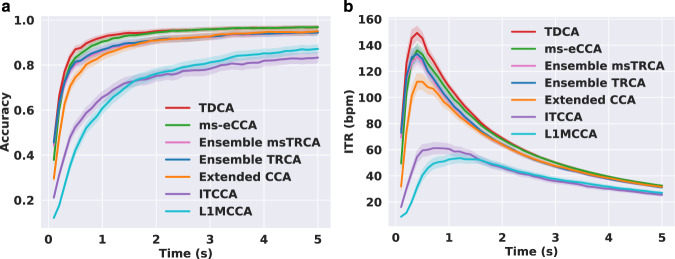
The average classification accuracy (**a**) and ITR (**b**) for the seven different supervised methods. Data lengths from 0.1 s to 5 s with an interval of 0.1 s were used for evaluation. The shaded area around the curve denotes the standard error of the mean. (Best view in color).

For the supervised methods, we evaluated the data records by comparing seven methods, including task-discriminant component analysis (TDCA)^53^, multi-stimulus extended CCA (ms-eCCA)^54^, ensemble multi-stimulus task-related component analysis (ensemble msTRCA)^54^, ensemble task-related component analysis (ensemble TRCA)^14^, extended canonical correlation analysis (Extended CCA)^55^, individual template-based CCA (ITCCA)^56^, and L1-regularized multiway CCA (L1MCCA)^57^. Specifically, for each participant, leave-one-block-out cross validation was conducted for evaluation. For TDCA, the parameters of $${N}_{k}=5$$, $$l=3$$ were set, which was based on the parameter selection in Supplementary Figure 2. The filter-bank technique was applied in TDCA, ms-eCCA, ensemble msTRCA, ensemble TRCA and Extended CCA, with the number of filter banks $${N}_{fb}=5$$ and the weights were set according to the previous study^16^. The procedure of filter bank filtering was conducted for each data length. The results of the average classification accuracy and ITR were illustrated in Fig. 5. As assessed by one-way repeated measures analysis of variance (RMANOVA), there was a significant difference among methods for all data lengths in the accuracies and ITRs, with all $$p < 0.001$$. For a short data length of 0.4 s, post-hoc pairwise comparisons revealed that TDCA > ms-eCCA/ensemble TRCA/ensemble msTRCA > Extended CCA > ITCCA > L1MCCA on ITR, where “>” indicates a statistical significance $$ < 0.05$$ between the two sides after Bonferroni correction. For a medium data length of 1 s, the accuracies for the methods were as follows: TDCA: $$0.925\pm 0.012$$; ms-eCCA: $$0.904\pm 0.013$$; ensemble TRCA: $$0.868\pm 0.018$$; ensemble msTRCA: $$0.864\pm 0.018$$; Extended CCA: $$0.862\pm 0.017$$; ITCCA: $$0.657\pm 0.026$$; L1MCCA: $$0.608\pm 0.025$$. The highest ITRs were achieved at different data lengths for the methods and the results were as follows: TDCA: $$149.38\pm 5.28$$ bpm at 0.4 s; ms-eCCA: $$136.06\pm 5.15$$ bpm at 0.4 s; ensemble TRCA: $$133.36\pm 5.93$$ bpm at 0.4 s; ensemble msTRCA: $$131.15\pm 5.99$$ bpm at 0.4 s; Extended CCA: $$117.46\pm 5.60$$ bpm at 0.5 s; ITCCA: $$61.15\pm 4.33$$ bpm at 0.8 s; L1MCCA: $$53.58\pm 3.44$$ bpm at 1.2 s. Besides, we also evaluated other combined methods, e.g., ms-eCCA+ms-eTRCA^54^, and the performance comparisons between TDCA and ms-eCCA+ms-eTRCA or ms-eCCA were shown in Supplementary Figure 3 and 4, respectively. The result of the supervised methods revealed an average ITR of approximately up to 150 bpm at 0.4 s, which could satisfy the eldercare scenarios that demand high-speed output of commands.

**Fig. 6:**
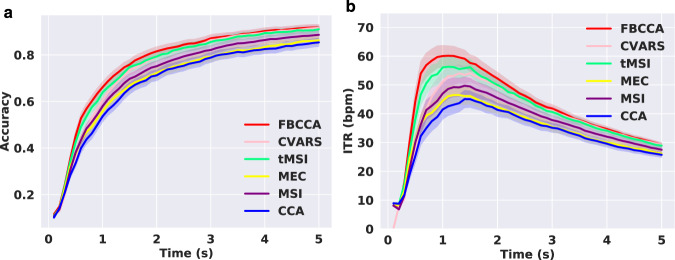
The average classification accuracy (**a**) and ITR (**b**) for the six different training-free methods. Data lengths from 0.1 s to 5 s with an interval of 0.1 s were used for evaluation. The shaded area around the curve denotes the standard error of the mean. (Best view in color).

In parallel, six training-free methods including filter bank CCA (FBCCA)^16^, canonical variates with autoregressive spectral analysis (CVARS)^58^, temporally local multivariate synchronization index (tMSI)^59^, minimum energy combination (MEC)^60^, multivariate synchronization index (MSI)^61^ and CCA^15^ were compared. For the methods except FBCCA, a band-pass filtering with a passband of 6 Hz∼100 Hz was applied. Figure 6 illustrates the average classification accuracy (**a**) and ITR (**b**) for the training-free methods. A significant difference between methods was found in the accuracies and ITRs, as revealed by one-way RMANOVA, with all $p < 0.001$. For a medium data length of 1 s, post-hoc pairwise comparisons revealed that FBCCA > tMSI > CVARS/MSI > MEC/CCA on ITR. For a long data length of 5 s, the accuracies for the methods were as follows: FBCCA: $0.917\pm 0.013$; CVARS: $0.916\pm 0.013$; tMSI: $0.908\pm 0.014$; MSI: $0.89\pm 0.015$; MEC: $0.863\pm 0.017$; CCA: $0.854\pm 0.018$. The highest ITR for the training-free methods were attained after 1 s, and the results were as follows: FBCCA: $60.14\pm 3.44$ bpm at 1.1 s; tMSI: $56.58\pm 3.72$ bpm at 1 s; CVARS: $54.05\pm 2.78$ bpm at 1.5 s; MSI: $51\pm 3.3$ bpm at 1.2 s; MEC: $46.5\pm 3.08$ bpm at 1.3 s; CCA: $45.08\pm 3.0$ bpm at 1.4 s. The training-free methods suggest an average ITR up to 60 bpm and 5-s accuracy above 90 %, which suits the eldercare scenarios that require no calibration for plug-and-play BCI control.

Furthermore, we calculated the accuracies for different stimulus frequencies and the result was shown in Supplementary Figure 5. Here we evaluated the 13 methods by the data records with a data length of 5 s, and the classification accuracies were averaged across all the methods. As assessed by one-way RMANOVA, there was a statistically significant difference in accuracies for different stimulus frequencies, $F(4.186,476.811)=3.689,p < 0.001$. The result also indicated a marginally significant tendency of decline in accuracy as the stimulus increased, $r=-0.065$, $p=0.051$, which was consistent with the tendency of decrease in SNR as the stimulus frequency increased in Fig. 3(a). In addition, the change in the maximum average ITR with blocks was further analyzed and the result was shown in Supplementary Figure 6. The ITR values were averaged across the six training-free methods. As revealed by one-way RMANOVA, there was a statistically significant difference in ITRs for different blocks, $F(5.573,551.74)=2.783$, $p=0.013$. During the course of blocks, there was a tendency of a slight decrease in the ITRs, though not statistically significant, $r=-0.056$, $p=0.139$.

### Power law analysis

**Fig. 7:**
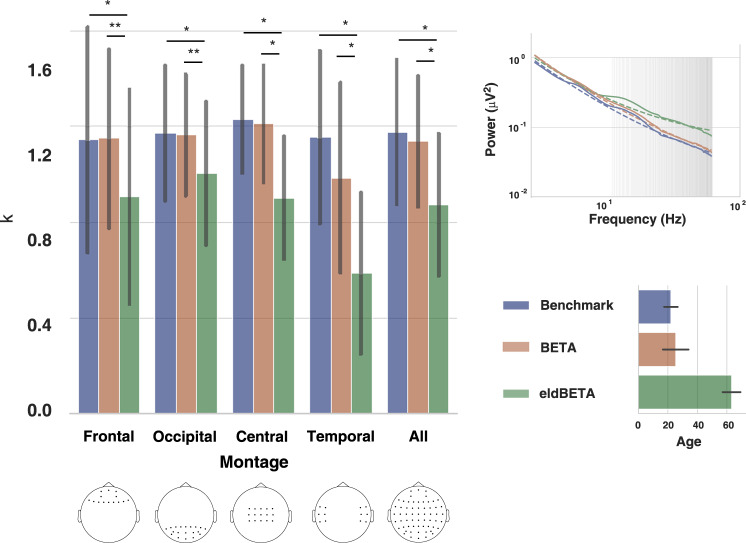
The comparison of the power-law characteristics for the eldBETA, the BETA, and the Benchmark databases. (Upper left) Bar plot of the negative of the power-law exponent for the three databases with different montages. (Bottom left) The channel location corresponding to each montage. The asterisks indicate a statistically significant difference between the pairs. *$p < 0.05$, **$p < 0.01$, ***$p < 0.001$, Bonferroni corrected. (Upper right) The power spectrum (solid curves) with the associated power-law fitting (dashed curves) for the three databases. The shaded area indicates a statistical significance ($p < 0.05$) between the three databases as assessed by one-way RMANOVA. Green: the eldBETA database; Brown: the BETA database; Blue: the Benchmark database. (Bottom right) Bar plot of the age distribution for the three databases. (Best view in color).

Besides the narrow-band oscillation of SSVEPs, we further validated the broadband fractal characteristics of the data records by quantifying the power-law exponent of the spectrum. Here, a spectral separation method, namely the irregular-resampling auto-spectral analysis (IRASA)^62^ was used to extract the scale-free fractal signals. Specifically, the 0∼3 s of the SSVEPs from 5 stimulus frequencies (8 Hz, 9 Hz, 10 Hz, 11 Hz, and 12 Hz) were analyzed by IRASA, in which the frequency range for spectral separation and power-law fitting was from 3 Hz to 100 Hz. By means of the power-law fitting, we estimated the power-law exponent, a.k.a. the slope factor of the linear model that fitted the power spectrum in the log-log plot. For each of the 64 channels, the power-law exponent was computed and then averaged by blocks and by conditions. Finally, the exponent values were averaged by channels from the region of interest (ROI), in which five typical montages (frontal, occipital, central, temporal and all channels, details were listed in Table 3) were evaluated. This procedure was applied to the Benchmark database as well as the BETA database for comparison. In the BETA database, since the duration of visual stimulation for S1∼S15 was 2 s, these participants were excluded for analysis and participants from S16 to S70 with a stimulation duration of 3 s were evaluated. The negative of the power-law exponential ($k$) for the three databases was illustrated in Fig. 7. For each montage, Student’s *t* test with Bonferroni correction revealed that the exponent *k* in the eldBETA was significantly smaller than that in the Benchmark database and than that in the BETA database, with all $p < 0.001$. For instance, for the three databases in the montage of all channels, the average power spectrum (solid curves) with the associated power-law fitting (dashed curves) was illustrated in Fig. 7 (upper right panel) and the average exponent for each database was as follows: eldBETA: $0.872\pm 0.301$; Benchmark: $1.176\pm 0.307$; BETA: $1.137\pm 0.277$. Compared with the Benchmark and BETA database, the lower value of the exponent in eldBETA indicates an increase in the broadband noise. Since the participants from the eldBETA database have older ages (illustrated in the bottom right panel), the result of the power-law analysis is then in line with the previous study^63^, and lends support to the neural noise hypothesis of aging^64,65^.

**Table 3:** The comprised channels of four typical montages.

Frontal	FP1/2, AF3/4, FPz, Fz, F1/2. F3/4, F5/6, F7/8
Central	FC3/4, Cz, C1/2, C3/4, CPz, CP1/2, CP3/4
Temporal	FT7/8, FC5/6, T7/8, C5/6, CP5/6, CP7/8
Occipital	Pz, P1/2, P3/4, P5/6, P7/8, POz, PO3/4, PO5/6, PO7/8

### BCIQ profile

**Fig. 8:**
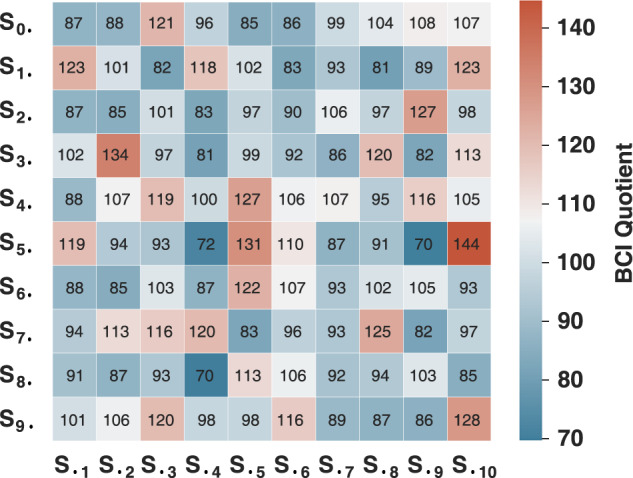
The BCI quotient (BCIQ) for each participant. Participants were indexed from S1 to S100 (S100 could be indexed by S_9·_ and S_·__10_). Each row denotes an individual and each column denotes tens of individuals. Warmer color indicates a higher BCIQ and cooler color indicates a lower BCIQ. The value in the square denotes the BCIQ of the participant. (Best view in color).

To validate the individual difference and variety in the data records, the metric of the BCIQ for each participant was calculated in Eq. (5) and then displayed in Fig. 8. Here, each participant (S1∼S100) is indexed by a row and a column, where a row denotes an individual and a column denotes tens of individuals. In line with the previous study^29^, the BCIQ for the data records can well predict the proficiency in using the BCI, i.e., the BCI performance, with $r=0.768$, $p < 0.001$. For instance, participant S1 and participant S100 have the BCIQ of 87 and 128, respectively, which reveals the projected low (20 bpm, maximum average ITR of FBCCA) and high (158 bpm, maximum average ITR of FBCCA) proficiency for the BCI speller.

## Usage Notes

The following key notes are provided for better usage of the data records.

- *Data import* Data can be imported to the workspace by loading the epoch data record in MATLAB, or using the package “scipy.io.loadmat” or “h5py.File” in Python. For conventional classification analysis, the epoch data in the record are recommended. For other research purposes, e.g., asynchronous classification analysis, or blind source separation (BSS), the raw data without epoching and processing (the “.edf” data) could be utilized. The data structure of the “.edf” data is generated by the “loadcnt” and “writeeeg” function from EEGLAB, and the details can be referred to the EEGLAB. The epoch data can be extracted from the raw data.
- *Subset selection* A subset of the participants or the stimulus frequencies could be selected in the design of a specific BCI system. For instance, the BCIQ listed in Fig. 8 could serve as a guideline to select participants for developing a BCI system designated for a particular user population, e.g., BCI illiteracy.
- *Data partition* The cross validation that leaves one block out in each fold is a common practice in classification analysis. In the cross validation, the data partition by trials should be avoided due to the fact that there exist temporal correlations between trials in a block^66^, different from the data nature in other domains, e.g., computer vision. Also, a sliding window with a random onset in the test data should be avoided.

## Supplementary information

Supplementary figures for the manuscript.

## Data Availability

Custom codes for generation and processing of the data and the figures are presented in the repository^67^. A MATLAB script “eldbeta_convert.m” was provided for data processing in converting the raw data to the epoch data. The data preprocessing and technical validations were conducted in MATLAB R2018b and Python 3.6.10. A “README.md” file was used for a brief description of the code in the code repository. The Benchmark database and the BETA database as well as the classification algorithms can be found in their corresponding repositories related to the papers, and thus they are not provided in this data descriptor.
