# Supplementary material for: eldBETA: A Large Eldercare-oriented Benchmark Database of SSVEP-BCI for the Aging Population
Source: Sci Data. 2022 May 31;9:252. doi: 10.1038/s41597-022-01372-9 (PMC9156785; doi:10.1038/s41597-022-01372-9)
Supplement: Supplementary file 1 — Supplementary figures for the manuscript. [file 41597_2022_1372_MOESM1_ESM.pdf]

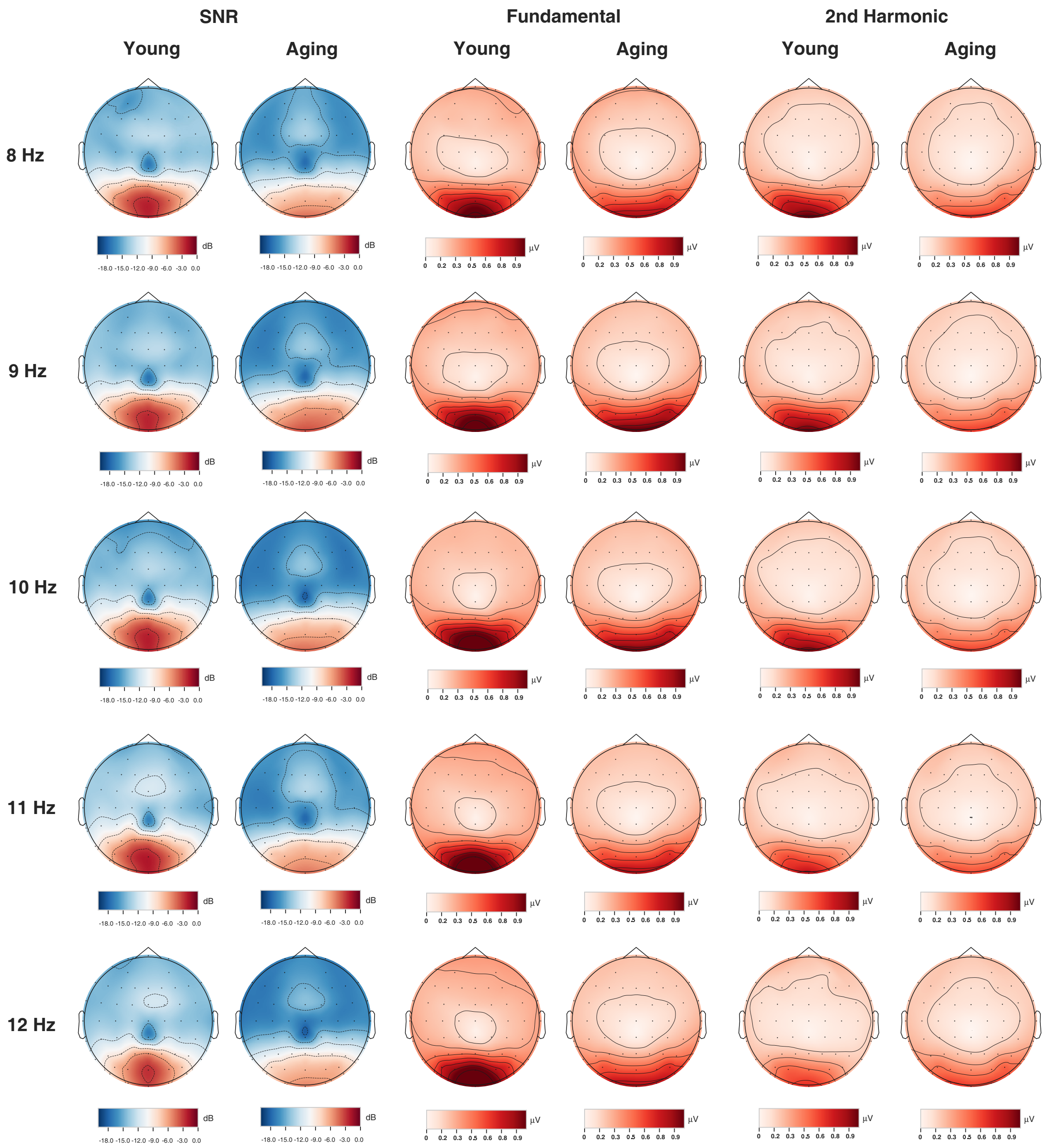

Figure 1. (Supplementary Figure 1) Topographic maps of average SNR and SSVEP amplitude for young participants (the Benchmark database) and aging participants (the eldBETA database) at different stimulus frequencies. For illustrative purpose, the spectral amplitude at the fundamental frequency and the 2nd harmonic are calculated and shown.

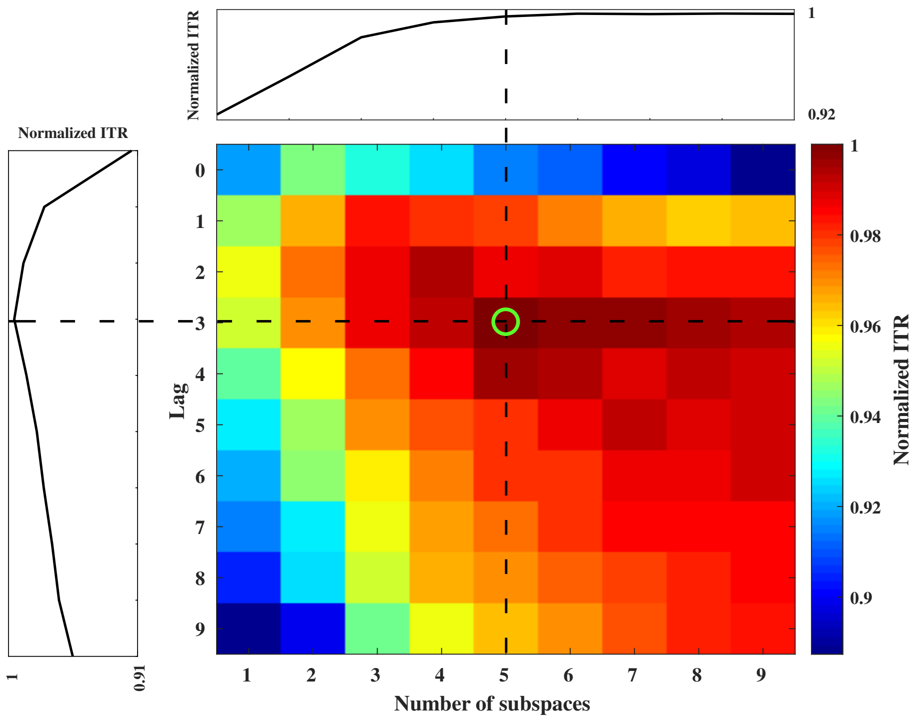

Figure 2. (Supplementary Figure 2) Normalized maximum average ITR of TDCA with a different number of subspaces and lag. The upper panel and the left panel are the ITR with a different number of subspaces and lag, respectively. The dashed line denotes the choice of parameters in this study. (Best view in color.)

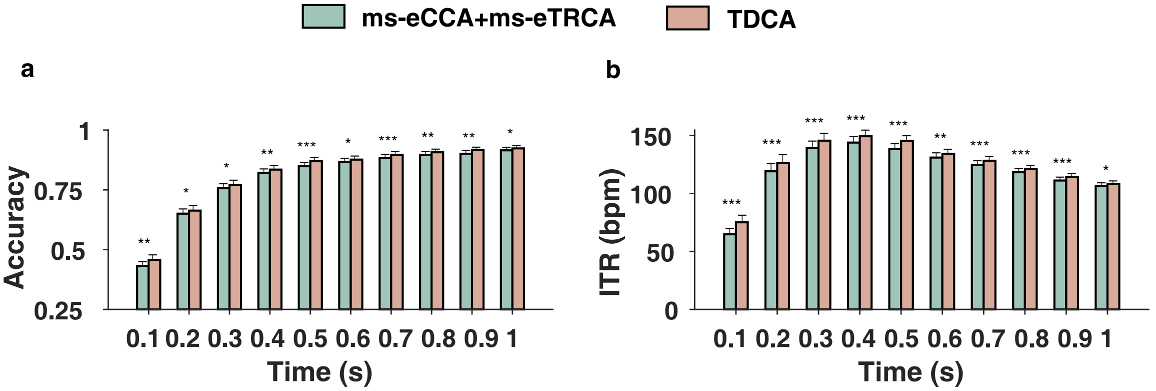

Figure 3. (Supplementary Figure 3) Average classification accuracy (a) and ITR (b) for the ms-eCCA+ms-eTRCA and the TDCA. Data lengths from 0.1 s to 1 s (with an interval of 0.1 s) are used for evaluation. The asterisks indicate significant differences between the ms-eCCA+ms-eTRCA and the TDCA by paired t-test ($*p < .05$, $**p < .01$, $***p < .001$).

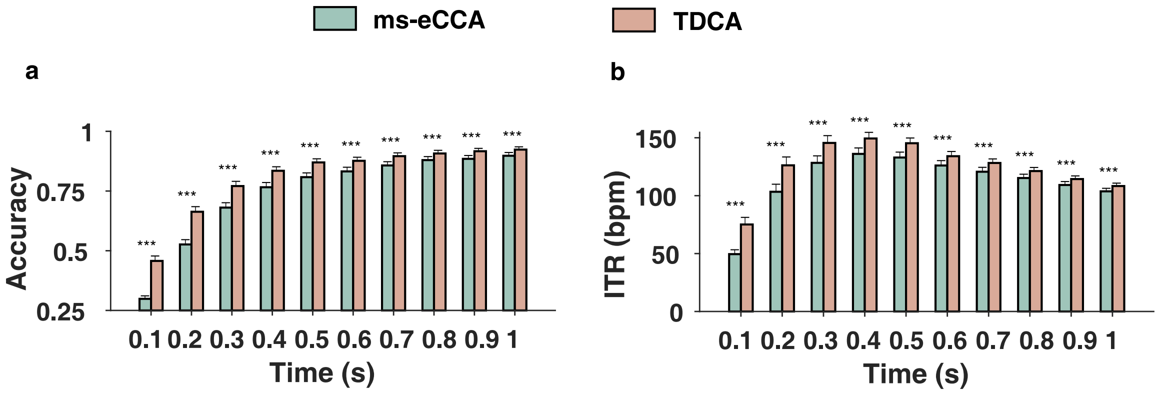

Figure 4. (Supplementary Figure 4) Average classification accuracy (a) and ITR (b) for the ms-eCCA and the TDCA. Data lengths from 0.1 s to 1 s (with an interval of 0.1 s) are used for evaluation. The asterisks indicate significant differences between the ms-eCCA and the TDCA by paired t-test ($*p < .05$, $**p < .01$, $***p < .001$).

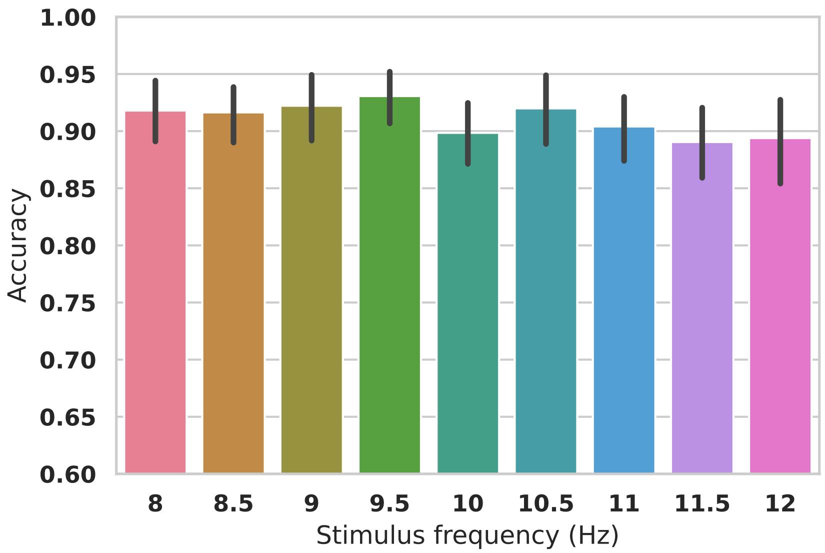

Figure 5. (Supplementary Figure 5) Average classification accuracies for different stimulus frequencies. Data length of 5 s was evaluated for the 13 target recognition methods and classification accuracies were averaged across all the methods.

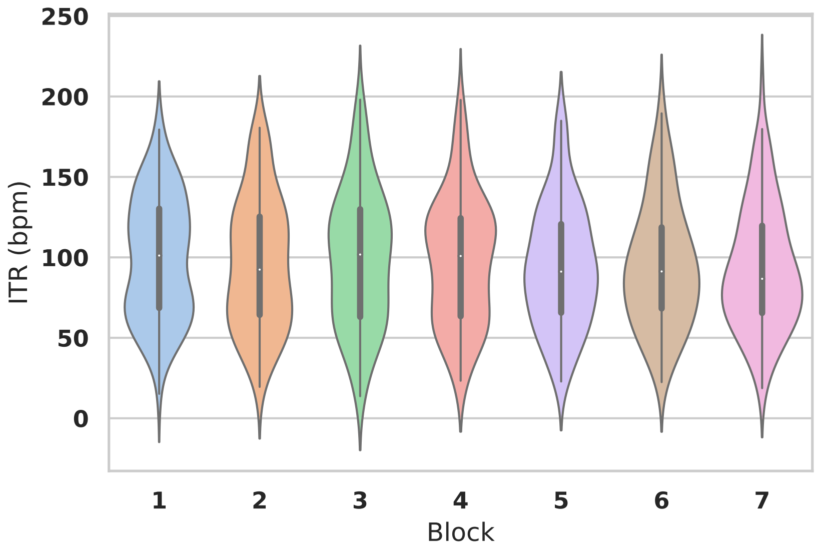

Figure 6. (Supplementary Figure 6) The change in maximum average ITR with respect to the block number in the experiment.
